# Subthalamic beta dynamics mirror Parkinsonian bradykinesia months after neurostimulator implantation

**DOI:** 10.1002/mds.27068

**Published:** 2017-06-22

**Authors:** Leon Amadeus Steiner, Wolf‐Julian Neumann, Franziska Staub‐Bartelt, Damian M. Herz, Huiling Tan, Alek Pogosyan, Andrea A. Kuhn, Peter Brown

**Affiliations:** ^1^ Department of Neurology, Charité, Campus Virchow Klinikum University Medicine Berlin Berlin Germany; ^2^ Nuffield Department of Clinical Neurosciences University of Oxford Oxford UK; ^3^ Medical Research Council Brain Network Dynamics Unit University of Oxford Oxford UK; ^4^ NeuroCure, Charité University Medicine Berlin Berlin Germany

**Keywords:** Parkinson's disease, bradykinesia, deep brain stimulation, local field potential

## Abstract

**Background:**

Exaggerated oscillatory activity in the beta frequency band in the subthalamic nucleus has been suggested to be related to bradykinesia in Parkinson's disease (PD). However, studies seeking correlations between such activity in the local field potential and motor performance have been limited to the immediate postoperative period, which may be confounded by a stun effect that leads to the temporary alleviation of PD deficits.

**Methods:**

Local field potentials were recorded simultaneously with motor performance in PD patients several months after neurostimulator implantation. This was enabled by the chronic implantation of a pulse generator with the capacity to record and transmit local field potentials from deep brain stimulation electrodes. Specifically, we investigated oscillatory beta power dynamics and objective measures of bradykinesia during an upper limb alternating pronation and supination task in 9 patients.

**Results:**

Although beta power was suppressed during continuously repeated movements, this suppression progressively diminished over time in tandem with a progressive decrement in the frequency and amplitude of movements. The relationship between changes within local field potentials and movement parameters was significant across patients, and not present for theta/alpha frequencies (5‐12 Hz). Change in movement frequency furthermore related to beta power dynamics within patients.

**Conclusions:**

Changes in beta power are linked to changes in movement performance and the sequence effect of bradykinesia months after neurostimulator implantation. These findings provide further evidence that beta power may serve as a biomarker for bradykinesia and provide a suitable substrate for feedback control in chronic adaptive deep brain stimulation. © 2017 The Authors. Movement Disorders published by Wiley Periodicals, Inc. on behalf of International Parkinson and Movement Disorder Society.

The diagnosis of Parkinson's disease (PD) is primarily clinical, with bradykinesia being a core diagnostic feature compromising motor function. The widely accepted Queen Square Brain Bank criteria for the diagnosis of PD define bradykinesia as “slowness of initiation of voluntary movement with progressive reduction in speed and amplitude of repetitive action.”^1^ These features discriminate the impairment of movement in PD from that in other related conditions.^2^, ^3^, ^4^

Parkinsonian bradykinesia has been linked to exaggerated oscillatory activity in the beta frequency band in the local field potential (LFP) activity recorded from the subthalamic nucleus (STN). Correlative evidence has taken 3 forms. First, beta activity recorded in the STN at rest in patients withdrawn from their medication has been correlated with UPDRS motor scores across patients.^5^, ^6^, ^7^, ^8^ Second, there have been correlations between change in beta activity with treatments such as levodopa or DBS or during voluntary movements and change in UPDRS motor scores with treatment or movement across patients.^9^, ^10^, ^11^, ^12^ Finally, there have been rare studies that have correlated beta levels with objective measures of motor impairment within patients.^13^ It is the latter that provide the strongest motivation for consideration of beta activity basis for feedback‐controlled adaptive DBS.^14^, ^15^, ^16^, ^17^

Yet with 1 exception, these correlative studies have hitherto been only performed through acute intraoperative or postoperative recordings and, although beta spectral peaks and their reactivity have been reported months and years after DBS implantation,^18^, ^19^, ^20^, ^21^ it is unknown if correlations persist many months after surgery. The extent to which acute postoperative recordings can be taken as representative of the chronic state is unclear given the stun effect whereby parkinsonism may be paradoxically temporally improved after operation, even in the absence of stimulation.^22^ Even in acute perioperative recordings, the vast majority of clinical and objective behavioral correlations with beta activity have only been reported at the group level.

The 1 study thus far that examined the relationship between STN LFP power and kinematic variables many months after electrode implantation failed to find significant correlations.^23^ This study of 9 participants correlated the change in LFP power with stimulation with the change in movement amplitude and frequency across patients. However, the correlations, although not significant after corrections for multiple comparisons, were still negative and appreciable (about −0.5 for LFP power over 11‐15 Hz and about −0.4 for power over 15‐30 Hz), suggesting that increases in beta activity might contribute to bradykinesia. Taken together, these studies indicate that the extent to which beta power might serve as a stable biomarker of bradykinesia over time in individual patients remains uncertain.

Adaptive DBS must be chronic to be successfully introduced into clinical practice, so it is crucial that beta activity is shown to continue to predict or correlate with objectively recorded bradykinesia a long time after electrode implantation. Moreover, such a correlation should be present within (as well as across) patients if beta‐controlled adaptive DBS is to be successful. Here we address these issues using a chronically implanted pulse generator with the capacity to record and transmit LFPs from DBS electrodes (Activa PC+S, Medtronic, Inc., Minneapolis, Minnesota). Specifically, we consider whether changes in beta activity might be related to the progressive reduction in speed and amplitude of repetitive action within a patient—a core component of bradykinesia that we term the *sequence effect*.

## Methods

### Patients and Surgery

A total of 15 patients with PD who underwent bilateral implantation of DBS electrodes in the STN were included in the study (see Supplementary Table 1). Informed consent was obtained before inclusion in the study, which was approved by the local ethics committee in accordance with the standards set by the Declaration of Helsinki. The DBS electrode used was model 3389 (Medtronic) connected to an Activa PC+S (Medtronic) implanted pulse generator. Correct placement of the DBS electrodes was confirmed by intraoperative microelectrode recordings and postoperative MRI in all patients. Contacts 0 and 3 were the lowermost and uppermost contacts, respectively. Contacts used to obtain bipolar recordings of the LFP reported in this study were the 2 contacts surrounding the contact, which had proven to be clinically most efficient in the months after neurostimulator implantation.

### Paradigm and Recordings

Most patients were recorded at 3 and 8 months following implantation surgery. Wherever possible, we used data recorded 8 months following implantation surgery so as to limit any confounds due to the stun effect. Where patients were not seen at 8 months or data from this assessment were considered suboptimal (see later) we analyzed data recorded at 3 months (see Supplementary Table 2). In practice, 6 of the included patients were studied at 8 months, the remaining 3 patients at 3 months after DBS surgery (see later). All patients were tested after a 12‐hour withdrawal from dopaminergic medication and with DBS turned off at least 30 minutes prior to the recording. During recordings, the patients were seated comfortably in an armchair and asked to perform pronation‐supination movements with the clinically more affected upper limb. Following a 30‐second block of rest recording, the patients were instructed to continuously rotate the handle of a rotometer^10^ as quickly and with the largest amplitude possible for 30 seconds. They could initiate and execute the movement sequence in their own time so that it was self‐paced in nature.

This was followed by another 30 seconds of rest recording before repeating the task with the same instructions. In total, 3 blocks of movement were recorded per session, resulting in a total recording lasting between 3 and 4 minutes. Short recording lengths were chosen to keep fatigue and battery discharge related to telemetric data transfer minimal.

LFPs were amplified (×2000), filtered at 1 to 100 Hz, and recorded at a sampling rate of 422 Hz onto the implanted pulse generator. Bipolar recordings were derived from the contacts immediately flanking the contact independently selected for chronic stimulation to mirror as closely as possible what would be performed during adaptive deep brain stimulation (aDBS). The optimal contact for chronic stimulation was the penultimate one in each case. All data were downloaded to a personal computer for offline analysis using telemetry. Movement performance was monitored using a custom‐made rotometer designed to track alternating pronation and supination movements.^10^ Angular rotometer deflection was converted to analog voltage and recorded using Spike 2 software (Cambridge Electric Design Limited, Cambridge, England). The sampling frequency of the rotometer was 422 Hz (same as for the electrophysiological recording).

Because LFPs and motor performance were recorded to different devices, they had to be synchronized offline. To synchronize LFP and movement recordings, stimulation (140 Hz) was briefly (< 3 seconds) switched on 2 or 3 times at the beginning of each session. This allowed offline alignment of the time series with respect to the stimulation artefact, which was present both in LFP and external recordings. To be more precise, we recorded the stimulation artefact externally with 2 electrodes, 1 placed on the midline of the scull and the other close to the implanted pulse generator. This allowed for the precise synchronization of both files (see Supplementary Fig. 1). The stimulation was then turned off throughout the session.

Offline analysis of LFP and movement data was performed using custom MATLAB (The Mathworks, Natick, Massachusetts) and Spike 2 (Cambridge Electric Design Limited) scripts. Power and phase of LFPs were computed using the continuous wavelet transform of the Morlet family with 6 cycles per frequency in 1‐Hz steps. Movement amplitude was taken from the Hilbert transform of the movement trace, previously bandpass filtered between 0.2 and 5 Hz. Movement frequency was calculated from zero crossings of the detrended movement trace.

### Patient/Hemisphere Selection

For each individual we chose 1 hemisphere for further analysis. The primary criterion for hemisphere selection was “movement‐induced beta reactivity,” defined as spectral power_movement_ − spectral power_rest_)/spectral power_rest_. Beta was operationally defined as 13 to 35 Hz. If both hemispheres showed movement reactivity (with >5% drop in power in the beta frequency band), the hemisphere contralateral to the movement performing upper limb was chosen. If only the contralateral or ipsilateral hemisphere showed movement reactivity, then the corresponding hemisphere was chosen for further analysis. If neither hemisphere showed movement reactivity in the beta frequency band, the respective patient was excluded (exclusion criterion I, 3 patients). Patients were also excluded when the synchronization procedure failed and the synchronization artefact was not reliably detectable in either LFP or movement traces (exclusion criterion II, 2 patients). Finally, patients with severe tremor off DBS interfering with task performance were excluded (exclusion criterion III, 1 patient). Application of the these exclusion criteria led to a preliminary exclusion of 6 of 12 patients who completed the 8‐month follow‐up (3 patients were excluded because they declined to undergo recordings off medication). By applying the same criteria to the patients who had completed the 3‐month follow‐up, we were able to include 3 patients who were rejected due to their 8‐month follow‐up findings (2 patients) or did not complete the 8‐month testing, but did complete the 3‐month follow‐up (1 patient). Together, this resulted in 9 hemispheres (6 contralateral, 3 ipsilateral to movement performing upper limb) from 9 individual patients in our analysis (Supplementary Table 2).

### Statistical Analyses

Statistical analyses were performed both in MATLAB (The Mathworks) and SPSS (IBM, Armonk, New York). For the analyses, both LFP power and motor performance were derived from the mean activity over 2 10‐second windows per 30‐second movement block. The 1st 10‐second window began at the beginning of the block, and the second window was taken to start 15 seconds after the beginning of the block. We choose to take a 15‐ instead of a 20‐second offset to ensure blocks with durations slightly less than 30 seconds could still be included. This allowed us to assess the change in motor performance over time and its relationship with changes in STN LFPs. The kinematic parameters (movement frequency and amplitude) of each window were normalized by subtraction of the patient‐specific mean of all 6 10‐second windows and compared using a 3 × 2 repeated‐measures analysis of variance with factors block (blocks 1, 2, and 3) and window (1st vs 2nd 10‐second window within each block). Whenever we found a violation of sphericity in Mauchly's sphericity test, Greenhouse‐Geiser corrections were applied.

In all further analyses, we considered change in both kinematic parameters and LFP dynamics as the percentage of change from the 1st to the 2nd 10‐second window within each block. To include data of all 3 blocks in across‐participant analyses, we applied regression analyses using change in the subthalamic oscillatory power as the predictor and change in the respective kinematic parameter (movement frequency or amplitude) as the dependent variable. We used a linear mixed‐effects regression model with blocks as repeated measures, in which the intercept was allowed to vary between blocks (random effect), and the slope of the regressions was treated as fixed effect across blocks. Based on previous reports,^10^, ^24^ we conducted these correlations for individual beta power (patient‐specific beta peak during movement performance, defined as the frequency bin with highest power in the beta band, ± 5Hz), changes in the predefined beta band (13‐35 Hz), and the predefined theta/alpha band (5‐12 Hz). The latter was chosen as a control frequency band to investigate frequency selectivity. To account for multiple comparisons, *P* values were corrected.^25^

Next, we conducted within‐participant correlation analyses by correlating changes in STN power with changes in kinematic parameters (1st vs 2nd 10‐second window within each block) across the 3 blocks recorded in each patient. To evaluate trends for individuals across blocks, we used nonparametric Spearman correlations because of the low number of observations (n = 3) per participant. Rho values were subsequently Fisher rho‐ to z‐transformed and compared to 0 using a 1‐sample, 2‐tailed *t*‐test. Means are reported in the form of mean ± standard error of mean.

## Results

**Figure 1:**
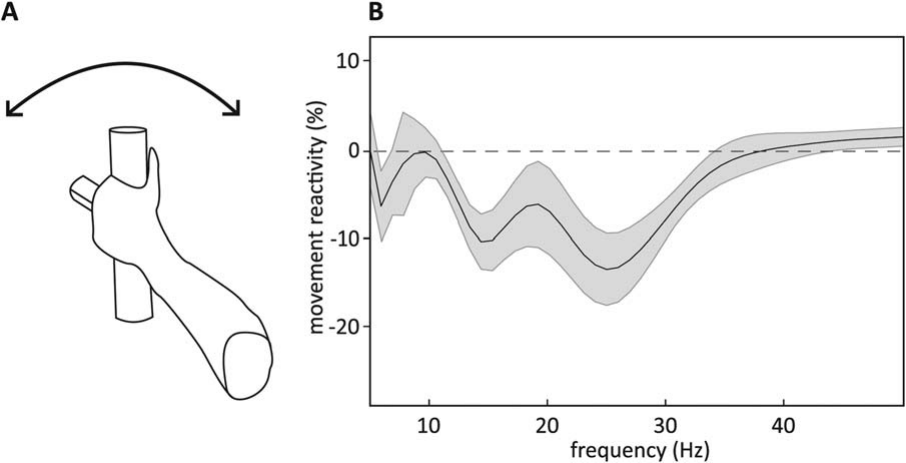
Suppression of oscillatory activity in a continuous alternating pronation and supination (rotometer) task months after neurostimulator implantation. (**A**) Schematic drawing of rotometer‐task paradigm. (**B**) Movement reactivity spectral data (calculated by subtracting rest from movement power spectral data and normalized by dividing by rest power spectral data taken from in between the 30‐second movement blocks) averaged over all 3 blocks of movement and then across the 9 participants. Shadow indicates standard error of mean. Averaging was performed irrespective of motor performance.

Bipolar recordings were derived from the contacts immediately flanking the contact independently selected for chronic stimulation. Suppression of oscillatory activity over the beta frequency band (13‐35 Hz) with 2 troughs during movements was observed in the LFP reactivity averaged across patients (Fig. 1).

**Figure 2:**
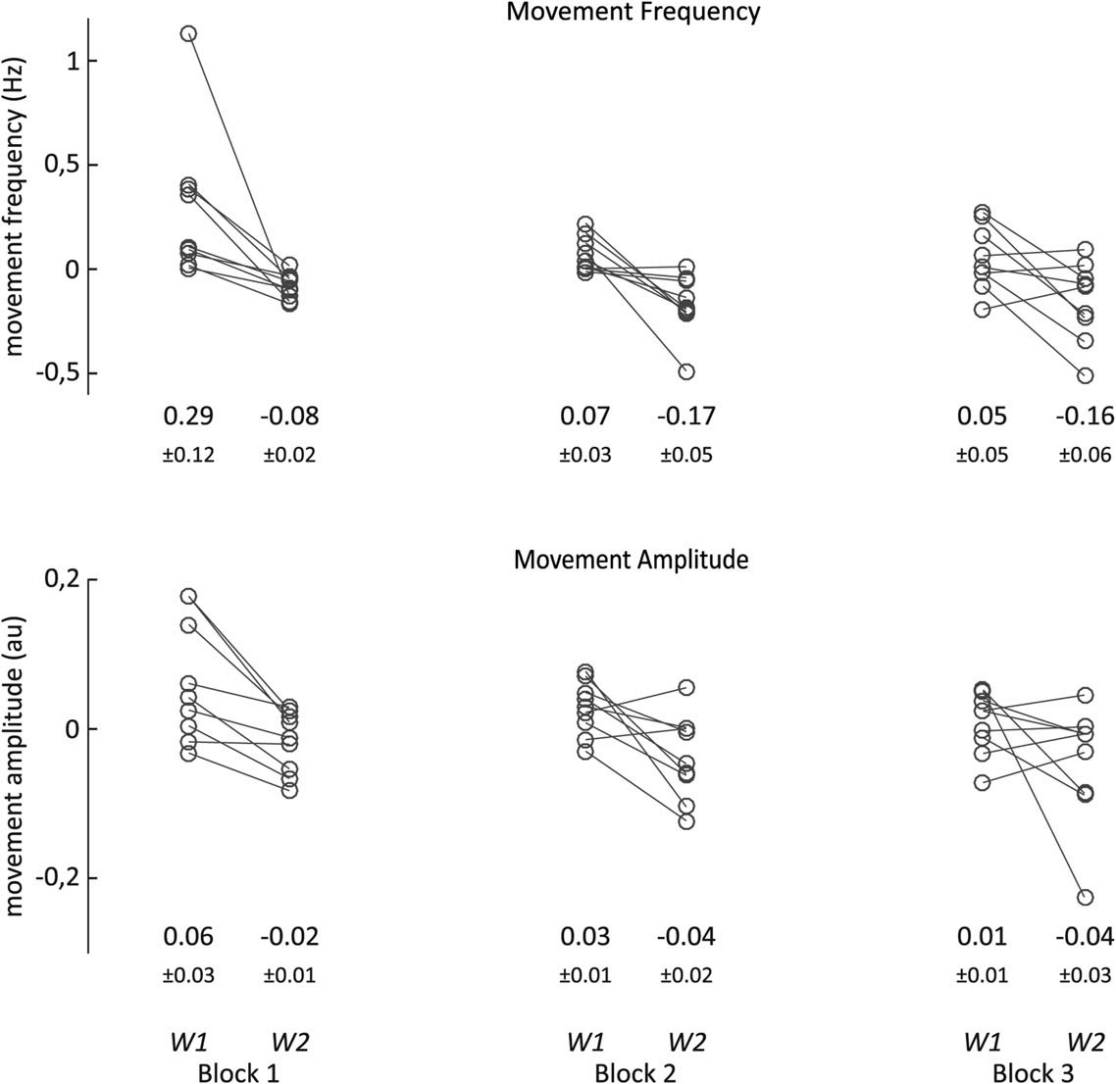
Changes in motor performance. Mean activity over the 2 10‐second windows (W1, window 1; W2, window 2) per block are displayed for each individual. Values were normalized by individual mean subtraction (see Methods). Below each window means + standard error of mean are given for the respective window. Analysis of variance revealed significant effect of window, no effect of block or window‐block interaction (see Results).

To track motor performance, we analyzed kinematic parameters, specifically movement amplitude and frequency (Fig. 2). The patient‐specific mean movement amplitude and frequency in each window was normalized by subtraction of the patient‐specific mean of all 6 10‐second windows. Analysis of variance revealed a significant decrease across patients in both kinematic parameters within blocks (main effect of window: movement frequency, *F*_1,8_ = 12.323, *P* = .008; movement amplitude, *F*_1,8_ = 8.754, *P* = .018), but no main effect of block (main effect of block: movement frequency, *F*_1.1,8.9_ = 2.628, *P* = .139; movement amplitude, *F*_2,16_ = 1.393, *P* = .277), or block × window interaction (movement frequency, *F*_1.2,9.7_ = 2.198, *P* = .17; movement amplitude, *F*_2,16_ = 0.841, *P* = .45) for either kinematic parameter. The main effect of window was due to a drop‐in movement amplitude and frequency between the 2 respective 10‐second windows within single blocks (mean of first windows = 0.136 ± 0.047; mean of second windows = −0.136 ± 0.027; data normalized by subtraction of patient‐specific mean of all 6 10‐second windows) and movement amplitude (mean of first windows = 0.033 ± 0.011; mean of second windows = −0.033 ± 0.012).

Thus, within blocks there was a sequence effect that was compatible with bradykinesia. In the next step, we tested whether the observed decrements in kinematic parameters were related to cotemporaneous changes in STN beta power.

### Relationship Between Bradykinesia and STN Beta Power Dynamics

**Figure 3:**
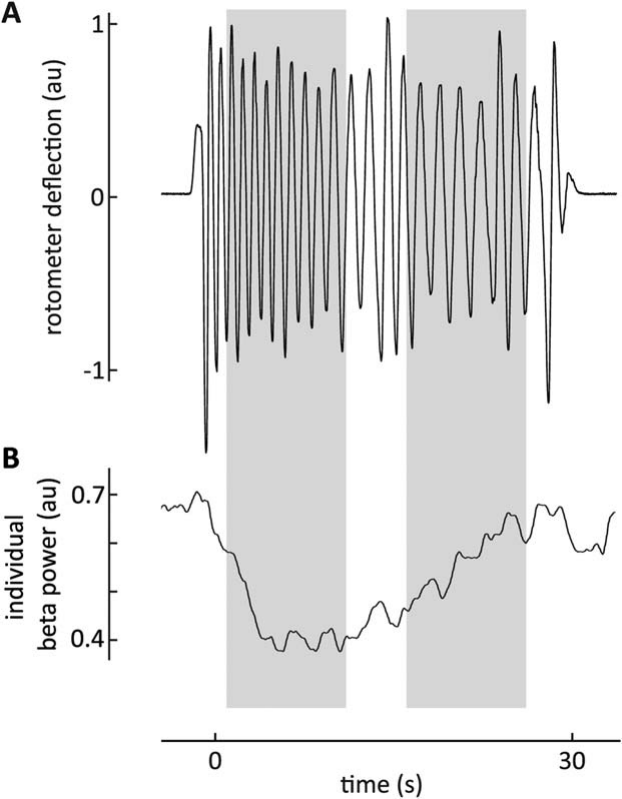
Example trace of beta power dynamics alongside motor impairment. Gray boxes indicate 10‐second windows, the means of which were used in further analysis. (**A**) Movement trace of a PD patient performing continuous and alternating pronation and supination movements for 30 seconds. Raw movement trace shown was detrended to allow better assessment of movement amplitude. (**B**) Trace of individual beta power (patient‐specific beta peak during movement performance ± 5 Hz) smoothed using an overlapping, sliding average window to capture the general trend in beta activity over time. Smoothing was applied for visualization purposes only. Unlike Figure 1, beta power is not normalized by rest power.

**Figure 4:**
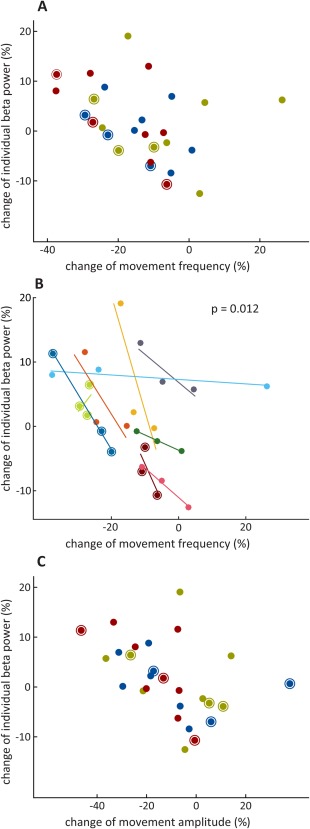
Relationship between change in individual beta power and change in movement characteristics across and within participants. The latter were frequency (**A** and **B**) and amplitude (**C**). Individual beta power was defined as patient‐specific beta peak during movement performance ± 5 Hz. All 3 blocks of performance by each respective individual are displayed. Circled data points were taken from ipsilateral hemispheres. Across the participant analysis (**A** and **C**), red = 1st 30‐second block of movement; blue = 2nd 30‐second block of movement; khaki = 3rd 30‐second block of movement. Within‐participant analysis (**B**): each color accounts for 1 individual. Rho values taken from Spearman's correlation for each individual were Fisher‐rho‐to‐z‐transformed and compared against 0 using a 1‐sample, 2‐tailed *t*‐test to arrive at the *P* value shown.

An example of concomitant changes in behavioral performance and STN beta power is illustrated in Figure 3, showing that the movement‐induced suppression of beta power fell off in tandem with decrement in motor performance. Separate linear mixed‐effects regression models with blocks as repeated measures and movement frequency or amplitude as the dependent variables demonstrated significant relationships between the amplitude of individually defined beta peaks and movement frequency and amplitude (Fig. 4A,C and Table 1). A rise in beta power during a movement block was associated with a drop in movement amplitude or frequency. This was also true of mean amplitude in a predefined beta band of 13 to 35 Hz in the case of movement amplitude (Table 1). The relationship with beta activity was frequency selective in so far as there was no effect of LFP power in the theta/alpha band (Table 1).

**Table 1:** Results of linear mixed‐effects regression model

Predictor	Estimate of fixed slope	*F* value	*P* value
Dependent variable: change in movement frequency			
Predictor: change in individual beta	−0.842 ± 0.294	*F*_1,21_: 8.223	.018
Predictor: change in predefined beta	−0.870 ± 0.470	*F*_1,24.5_: 3.433	.113
Predictor: change in predefined theta/alpha	0.181 ± 0.208	*F*_1,21.2_: 0.758	.394
Dependent variable: change in movement amplitude			
Predictor: change in individual beta	−1.163 ± 0.360	*F*_1,16.8_: 10.459	.018
Predictor: change in predefined beta	−1.705 ± 0.565	*F*_1,18_: 9.111	.018
Predictor: change in predefined theta/alpha	0.315 ± 0.282	*F*_1,22.2_: 1.249	.331

Estimates of fixed slope (EFS) are reported in the form of EFS ± standard error of EFS. Individual beta: patient‐specific beta peak during movement performance ± 5 Hz; predefined beta: 13‐35 Hz; predefined theta/alpha: 5‐12 Hz. All *P* values shown are corrected for multiple comparisons.

These data documented a relationship between bradykinesia and change in STN beta power across the participants. To determine whether the same could be shown within participants, we considered the kinematic and power changes between blocks during the 3 repetitions of the task performed in each participant. A greater decrease in movement frequency in a given repetition was associated with a steeper increase in beta power within that repetition (mean ρ = −0.667, *t*_9_ = −3.245, *P* = .012). All but 1 patient demonstrated this direction of effect (Fig. 4B). No such relationship could be shown for change in movement amplitude at the within‐participant level (mean ρ = −0.06, *t*_9_ = −0.02, *P* = .985).

## Discussion

As expected from studies of separate single movements,^24^ beta activity in the STN LFP was also suppressed during repeated movements. Critically, however, the degree of suppression progressively diminished over time in tandem with a decrement in the frequency and amplitude of repetitive movements. This relationship was absent over theta/alpha band frequencies in line with the inconsistent relationship between such activity in the STN LFP and force generation.^26^ The change in movement amplitude and frequency was predicted, in terms of correlation, by the strength of STN beta activity across the participants, and the change in movement frequency could also be related to beta power dynamics within the participants. These findings are important because they suggest that the correlation between beta activity in the STN and the sequence effect of bradykinesia seen across patients in the acute postoperative period is maintained months after neurostimulator implantation. Only 1 other study has so far examined the relationship between STN LFP power and kinematic variables months after electrode implantation. Although this study failed to find significant correlations, the latter were still negative and appreciable (about −0.5 for LFP power over 11‐15 Hz and about −0.4 for that over 15‐30 Hz).^23^

The current study provides further evidence that beta activity may scale with motor impairment within each patient, and does so over months. The evidence that correlations might also exist within patients has hitherto been scant and limited to immediate postoperative recordings.^13^ Our findings help address concern about what might happen to beta‐responsive aDBS systems during movement, when beta activity is suppressed.^27^ It seems that even during repeated movements as here, or during sustained contractions,^12^ beta activity scales with measures of bradykinesia. Interestingly, this was true of both ipsilateral and contralateral STN beta activity in our relatively small sample, in line with the relative lack of lateralization evident in the STN in this frequency band.^28^ This lack of coarse lateralization would be against, but not preclude, a dominant STN; however, our sample was too small to explore this possibility.

Together, these findings further motivate consideration of beta power in the STN as a biomarker for bradykinesia components and a suitable substrate for feedback control in chronic aDBS. Indeed, we took care to record the LFP in a similar way to that in clinical aDBS studies.^14^, ^15^, ^16^, ^17^ Hence recordings were bipolar and involved the contacts neighboring the contact that was the most clinically effective during neurostimulation.

Our study was correlative and hence cannot be taken as evidence of a mechanistic role for excessive beta activity in motor impairment. Nevertheless, our findings remain relevant to the development of aDBS systems that only rely on a correlation between beta and motor impairment and do not necessarily assume causality.^14^, ^15^, ^16^, ^17^ Beta activity in these systems is merely used as a marker of when to stimulate or how much to stimulate.^17^

Although the change in movement frequency could be related to beta power dynamics within (and across) participants, this was not the case for decrements in movement amplitude within (as opposed to across) participants. The reason for this discrepancy may be that movement amplitude diminished through both bradykinesia and physiological fatigue across movement blocks. The latter has previously been shown to have substantial effects in age‐matched controls even during 15‐second sequences of finger tapping,^2^ and yet need not necessarily be reflected in climbing levels of beta activity in the STN.

It should also be noted that we could not detect beta band reactivity in all patients. There may be several technical, device‐related explanations for this in addition to possible targeting variance. The device used for recording has a relatively high noise floor,^29^ and the data recorded by it were prone to contamination by electrocardiographic artefact.^20^ This may further compromise the effective noise floor of recordings. Finally, we recorded from contacts flanking the contact that proved clinically most effective. Hence, we cannot exclude the possibility that beta activity might have been picked up using different contact derivations. In future applications of adaptive stimulation, one might have to think of a reasonable trade‐off between detecting the most robust beta feedback (optimizing feedback input) and stimulating at the best contact (optimizing stimulation output).

There therefore still remains the question of whether feedback from beta activity in the STN is sufficient for effective aDBS. For example, beta power in the STN correlates with bradykinesia and rigidity, but it does not do so with tremor.^7^, ^10^ Thus aDBS systems that rely on beta activity feedback run the risk of not controlling tremor. In practice, the sufficiency of the beta signal is a question that can only be addressed in large and chronic clinical studies. However, at least in small cohorts of acutely stimulated patients using analogue or binary (thresholded) feedback of beta levels has been shown to have similar efficacy (or better) to conventional continuous DBS, and yet to be more efficient and have fewer side effects.^14^, ^15^, ^16^, ^17^

## Author Roles

1) Research project: A. Conception, B. Organization, C. Execution; 2) Statistical Analysis: A. Design, B. Execution, C. Review and Critique; 3) Manuscript: A. Writing of the first draft, B. Review and Critique.

L.A.S.: 1B, 1C, 2A, 2B, 2C, 3A

W.‐J.N.: 1A, 1B, 1C

F.S.‐B.: 1B, 1C

D.M.H.: 2A, 2C, 3B

H.T.: 2A, 2C

A.P.: 2B, 2C

A.A.K.: 1A, 1B, 3B

P.B.: 1A, 1B, 2A, 2C, 3A

## Full financial disclosures for the previous 12 months

W.‐J.N. reports nonfinancial support from St. Jude, nonfinancial support from Ipsen Pharma, and nonfinancial support from Medtronic, outside the submitted work. A.A.K. reports grants from the German Research Foundation and DFG; nonfinancial support from Medtronic during the conduct of the study; grants and personal fees from Medtronic; personal fees and nonfinancial support from Boston Scientific; nonfinancial support from Ipsen Pharma outside the submitted work. P.B. has received honoraria from Medtronic and Boston Scientific. L.A.S., F.S.‐B., D.M.H., H.T., and A.P. have nothing to disclose.

## Supporting information

Additional Supporting Information may be found in the online version of this article at the publisher's web‐site.

Supplementary Information Figure 1

Supplementary Information Table 1

Supplementary Information Table 2
