# Supplementary material for: Subthalamic beta dynamics mirror Parkinsonian bradykinesia months after neurostimulator implantation
Source: Mov Disord. 2017 Jun 22;32(8):1183–90. doi: 10.1002/mds.27068 (PMC5575541; doi:10.1002/mds.27068)
Supplement: Supplementary file 2 — Supplementary Information Table 1 [file MDS-32-1183-s002.docx]

**Supplementary Material: Table 1**

| Subject | Age at neurostimulator implantation (years) | Sex | Disease duration (years) | Total UPDRS-III *** | Hand used/  scored | Upper limb Bradykinesia – UPDRS*** | Upper limb Tremor – UPDRS*** | Upper limb Rigidity- UPDRS*** |
| --- | --- | --- | --- | --- | --- | --- | --- | --- |
| 1 | 66 | M | 8 | 19.5** | right | 4 | 0 | 2 |
| 2 | 72 | M | 9 | 27** | left | 4,5 | 4,5 | 3,5 |
| 3 | 72 | F | 5 | 25** | right | 8 | 0 | 1 |
| 4 | 61 | F | 10 | 21.5** | right | 5 | 0 | 1,5 |
| 5 | 50 | M | 7 | 42* | right | 12 | 0 | 3 |
| 6 | 58 | F | 7 | 37** | right | 10 | 7,5 | 0 |
| 7 | 56 | M | 18 | 51.5* | left | 5,5 | 6 | 2,5 |
| 8 | 63 | M | 9 | 18* | right | 3 | 0 | 2,5 |
| 9 | 60 | M | 7 | 31.5** | right | 6,5 | 0 | 2 |

**Supplementary Material: Table 1. Clinical features of included patients.**

* 3 month follow up

** 8 month follow up

*** at recording time
