# Supplementary material for: Subthalamic beta dynamics mirror Parkinsonian bradykinesia months after neurostimulator implantation
Source: Mov Disord. 2017 Jun 22;32(8):1183–90. doi: 10.1002/mds.27068 (PMC5575541; doi:10.1002/mds.27068)
Supplement: Supplementary file 3 — Supplementary Information Table 2 [file MDS-32-1183-s003.docx]

| Subject | 3 month follow-up | 8 month follow-up | Hemisphere analysed |
| --- | --- | --- | --- |
|  | Not seen | Synchronization artefact not detectable in LFP | none |
|  | Synchronization artefact not detectable in LFP | Not seen | none |
|  | Synchronization artefact not detectable in LFP | Tremor contributing to performance | none |
|  | Synchronization artefact not detectable in LFP | No distinct beta reactivity either side | none |
| 1 | Synchronization artefact not detectable in LFP | ***Data included*** | Ipsilateral |
| 2 | Synchronization artefact not detectable in LFP | ***Data included*** | Contralateral |
|  | Synchronization artefact not detectable in LFP | No distinct beta reactivity either side | none |
| 3 | ***Data included*** | ***Data included*** | Contralateral |
| 4 | Synchronization artefact not detectable in LFP | ***Data included*** | Contralateral |
|  | No distinct beta reactivity either side | Not seen | none |
| 5 | ***Data included*** | Not seen | Ipsilateral |
| 6 | No distinct beta reactivity either side | ***Data included*** | Contralateral |
| 7 | ***Data included*** | No distinct beta reactivity either side | Ipsilateral |
| 8 | ***Data included*** | Synchronization artefact not detectable in LFP | Contralateral |
| 9 | ***Data included*** | ***Data included*** | Contralateral |

**Supplementary Material: Table 2**

**Supplementary Material: Table 2. Exclusion details.** Note that during the first half of the study the synchronization artefact recorded at 3 months was not detectable in the LFP. This was because initially the stimulation artefact for off-line synchronization was induced by transcutaneous electrical nerve stimulation (TENS). This procedure failed however as the synchronization artefact was not reliably detectable in the LFP trace and was subsequently changed so that the synchronization artefact was induced by briefly turning on DBS (see methods and Supplementary Material: Fig. 1). In the two subjects in whom data were recorded and who met inclusion criteria at 3 and 8 months, recordings consistently demonstrated increasing beta power from the beginning to the end of each trial, whilst movement frequency consistently decreased from the beginning to the end of each trial. This indicates that the within-subject correlation demonstrated in Fig. 4B was consistent over time. However, the sample size of two patients is too small to make any substantial claims in this regard.
